# Scrotal wall leiomyosarcoma: a case report and review of the literature

**DOI:** 10.1186/s13256-021-03076-x

**Published:** 2021-09-21

**Authors:** Zahra Erfani, Aileen Azari-yam, Seyed Reza Yahyazadeh

**Affiliations:** 1 Department of Urology, Shariati Hospital, Tehran University of Medical Sciences, Tehran, Iran; 2 Department of Pathology, Shariati Hospital, Tehran University of Medical Sciences, Tehran, Iran

**Keywords:** Scrotum, Leiomyosarcoma, Scrotal, Dartos muscle

## Abstract

**Background:**

Up to 30% of all scrotal masses are sarcomas. Leiomyosarcoma of the scrotal wall is rare, and its clinical significance and prognosis have not been well defined, since the most reported cases have little or no follow-up.

**Case presentation:**

We report a 45-year-old Caucasian man who was admitted with a firm, nontender, mobile scrotal wall mass from 15 months ago. Laboratory data including testicular tumor markers were within normal range, and transscrotal ultrasonography revealed an oval-shaped, hypoechogenic, solid mass with blood flow and well-defined border. Histopathologic examination and immunohistochemistry staining, following surgical excision, were in favor of malignant leiomyosarcoma.

**Conclusion:**

Here we describe the morphological features and immunohistochemical presentations of the tumor and the patient’s relatively long-term follow-up.

## Introduction

Up to 30% of all scrotal masses are sarcomas. Soft-tissue sarcomas of the genitourinary tract account for 2.1% of soft-tissue sarcomas in general and only 1–2% of urological malignancies [1]. The most common types of sarcomas are liposarcoma, leiomyosarcoma, rhabdomyosarcoma, undifferentiated pleomorphic sarcoma, and fibrosarcoma [2]. Prevalently, leiomyosarcomas originate from the spermatic cord (48%), testicular tunica (48%), epididymis (2%), and dartos muscle, as well as subcutaneous tissue of scrotal wall (2%).

These tumors can be seen in any age group but mostly in the sixth decade, and 80% of patients are above 40 years [3]. The clinical significance and course of leiomyosarcoma of the scrotal wall have not been well defined, since most reported cases have little or no follow-up. They are frequently mistaken for a benign condition, and the accurate diagnosis is revealed on histopathologic investigation.

Scrotal wall leiomyosarcoma not developing from the spermatic cord, epididymis, or testes is exceptional, and its clinical significance and prognosis have not been well defined. Here we describe the morphological features and immunohistochemical presentations of the tumor and its relatively long-term follow-up.

## Case report

A 45-year-old Caucasian man, a welder, was admitted to our hospital with the chief complaint of a painless right scrotal wall mass from 15 months ago, which had been, recently, increased in size. He denied any coexisting symptoms such as fever, chills, cough, dyspnea, nausea, vomiting, and diarrhea. He also denied any previous history of trauma, surgery, radiation exposure, and medications including anabolic steroids. Social history was positive for smoking tobacco use at 10 pack-years. His family history was negative for a similar condition. Initial physical examination on admission revealed body temperature 36.8 °C, heart rate 70 beats per minute, and, blood pressure 135/86 mmHg. Head and neck examination showed no evidence of lymphadenopathy. Heart and lung sounds were normal. Thorough abdominal examination findings were unremarkable, and the inguinal lymph nodes were not enlarged on palpation. Neurologically, the patient was completely normal. Genital examination showed a firm, nontender, mobile mass in the posterior wall of the right hemiscrotum, while penis, bilateral testes, epididymis, and the spermatic cords were clinically normal.

**Fig. 1:**
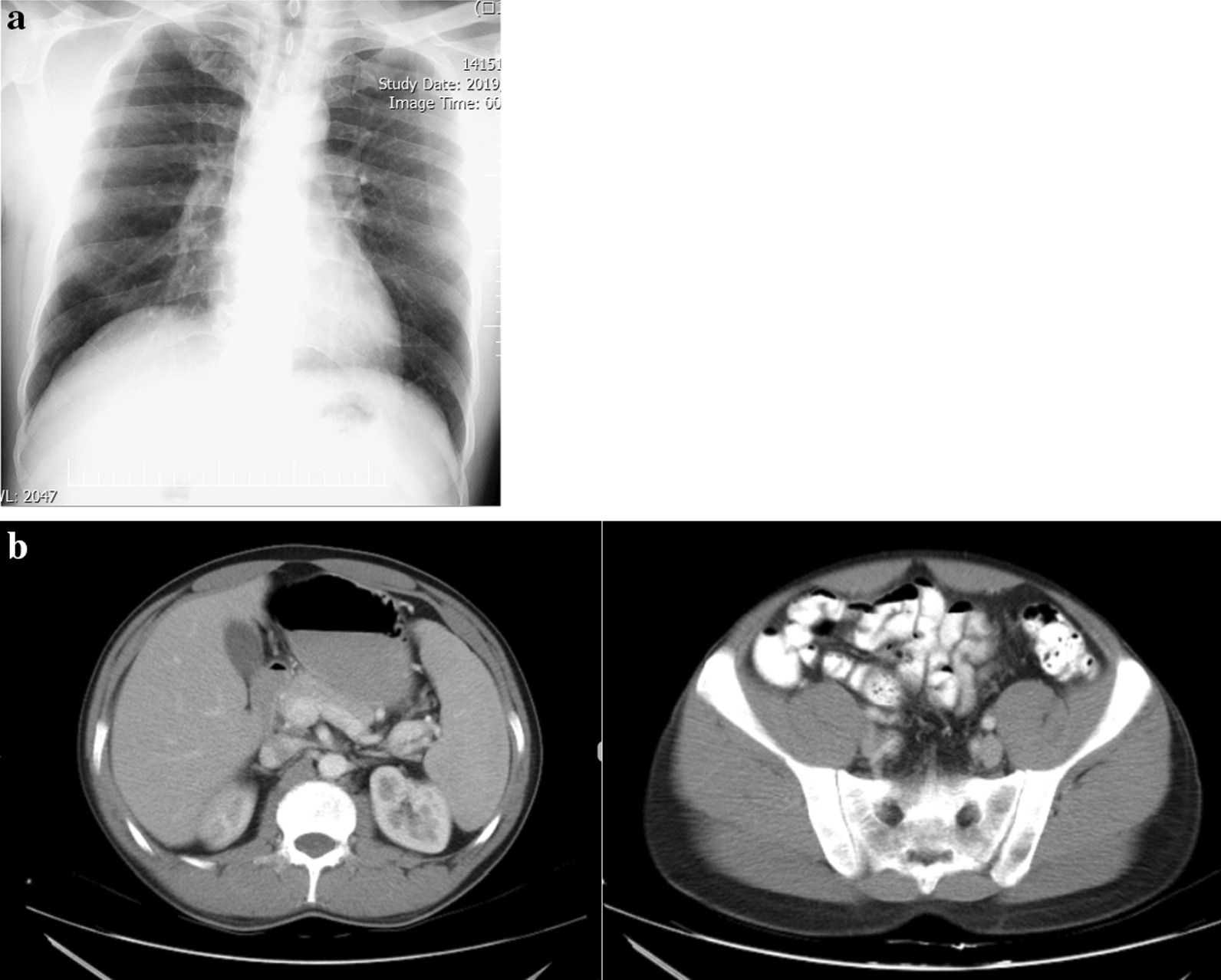
**a** Chest x-ray. **b** Computed tomography scan of the abdomen and pelvis showing no pathologic findings

Initial laboratory work-up included complete blood count with leukocyte count of 7200/μL, hemoglobin of 14.5 g/dL, and platelet count of 186,000/μL, renal function tests with blood urea nitrogen of 12.6 mg/dL and serum creatinine of 1.1 mg/dL; blood glucose of 99 mg/dL, erythrocyte sedimentation rate (ESR) of 12 mm/hour, and C-reactive protein (CRP) of 8 mg/L; biochemistry of Na 135 mEq/L, K 4.3 mEq/L, Ca 8.7 mg/dL, serum glutamic oxaloacetic transaminase (SGOT) 32 IU/L, serum glutamic pyruvic transaminase (SGPT) 36 IU/L, and alkaline phosphatase 214 IU/L. Urinalysis revealed no pathologic findings. The testicular tumor markers were within normal range: lactate dehydrogenase (LDH) 150 units/L, alpha-fetoprotein (AFP) 12 ng/mL, and beta-human chorionic gonadotrophin (β-HCG) less than 2 mIU/mL. Transscrotal ultrasonography revealed a 35 × 18 × 12-mm oval-shaped, hypoechogenic, solid mass with blood flow and well-defined border. Chest x-ray examination and abdominopelvic computed tomography (CT) scan were both normal, while whole-body bone scan showed no systemic metastasis (Fig. 1a, b).

**Fig. 2:**
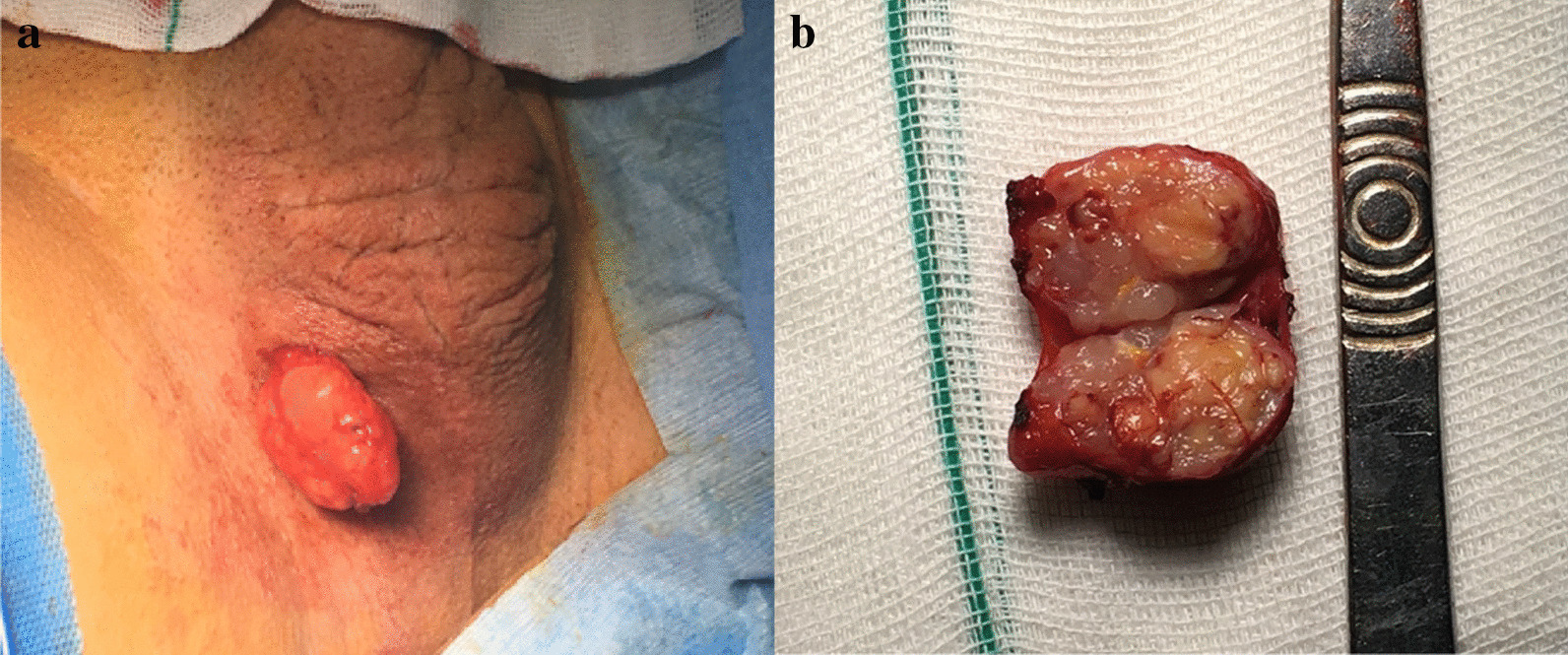
**a** The scrotal mass that is delivered through the skin incision. **b** Gross appearance of the mass with encapsulation, nodularity, and necrosis

Subsequently, the patient underwent an excisional biopsy, during which the mass was felt to be a 30 × 25 × 15-mm oval-shaped and well-circumscribed mass without capsule formation (Fig. 2a). Afterwards, the tumor was reported pathologically to be malignant spindle cell tumor, more probably of smooth muscle origin, which was attached to the surgical margins in some areas. Therefore, we did a second, wider marginal excision of the tumoral bed, which was reported to be free of residual tumors.

Preoperatively, we prescribed intravenous cefazolin (2 g) as the prophylactic antibiotic that was converted to oral cephalexin (500 mg per day) after discharge. The patient did not receive any neoadjuvant chemotherapy or radiation. Postoperative close follow-up was planned, and during the 20-month period, there was no evidence of recurrence or distant metastasis.

## Pathological finding

### Macroscopic findings

The specimen was an oval, encapsulated, solid, vaguely lobulated, creamy mass with a smooth surface measuring 28 × 25 × 10 mm. The cut surface was nodular and heterogeneous, consisting of regions with tan-yellow, gelatinous appearance and necrotic areas. Focally, the capsule was breached, and the tumor was touching the inked surgical margin. The subsequent margins showed no tumoral involvement (Fig. 2b).

### Microscopic findings

**Fig. 3:**
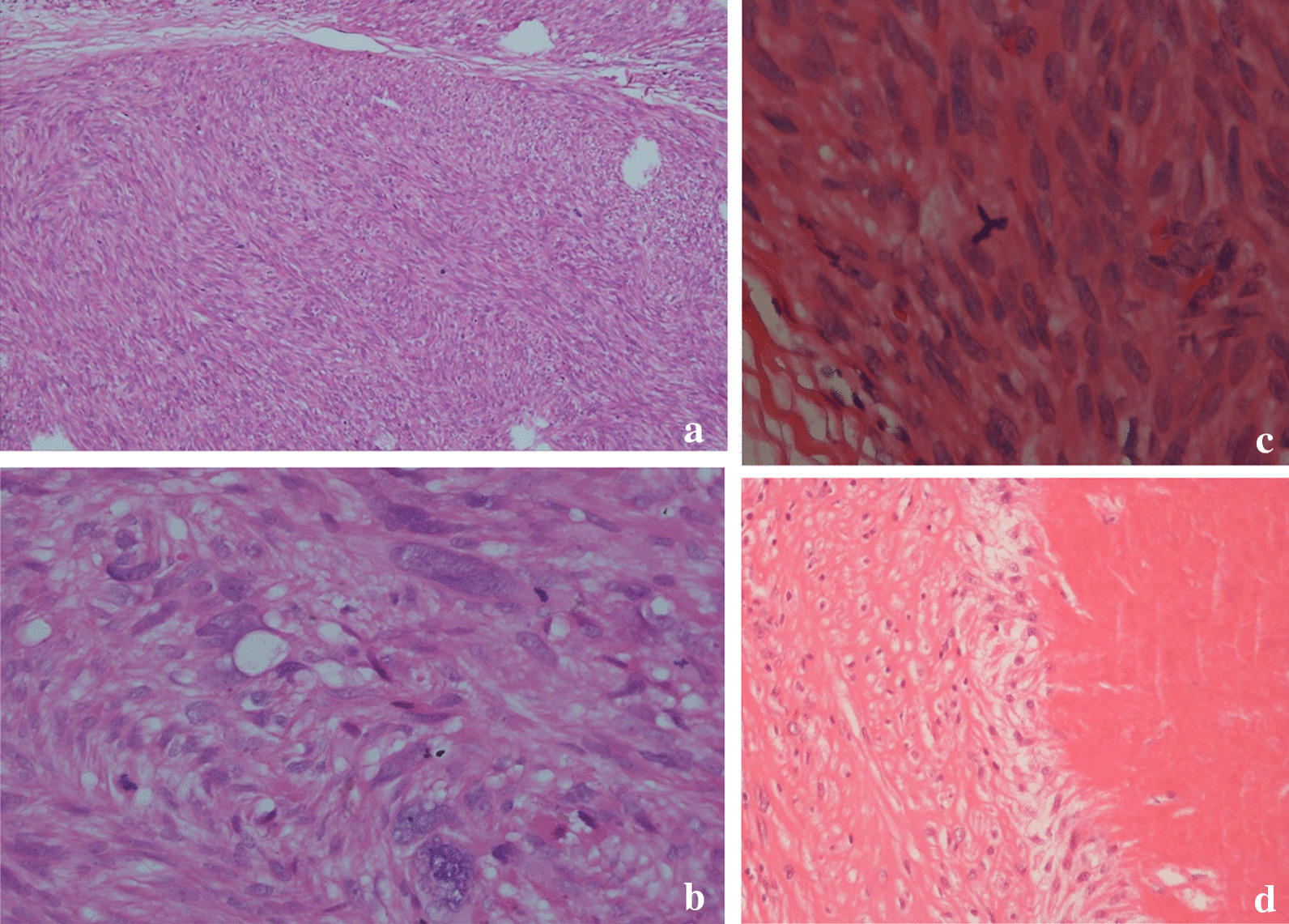
Microscopic view of the scrotal mass. **a** Spindle cell tumor with fascicular pattern and lobulation (hematoxylin–eosin stain, original magnification ×100). **b** Significant nuclear atypia (hematoxylin–eosin stain, original magnification ×400). **c** Atypical mitotic figure (hematoxylin–eosin stain, original magnification ×400). **d** Tumoral necrosis at right with palisading of tumor cells at its periphery

**Fig. 4:**
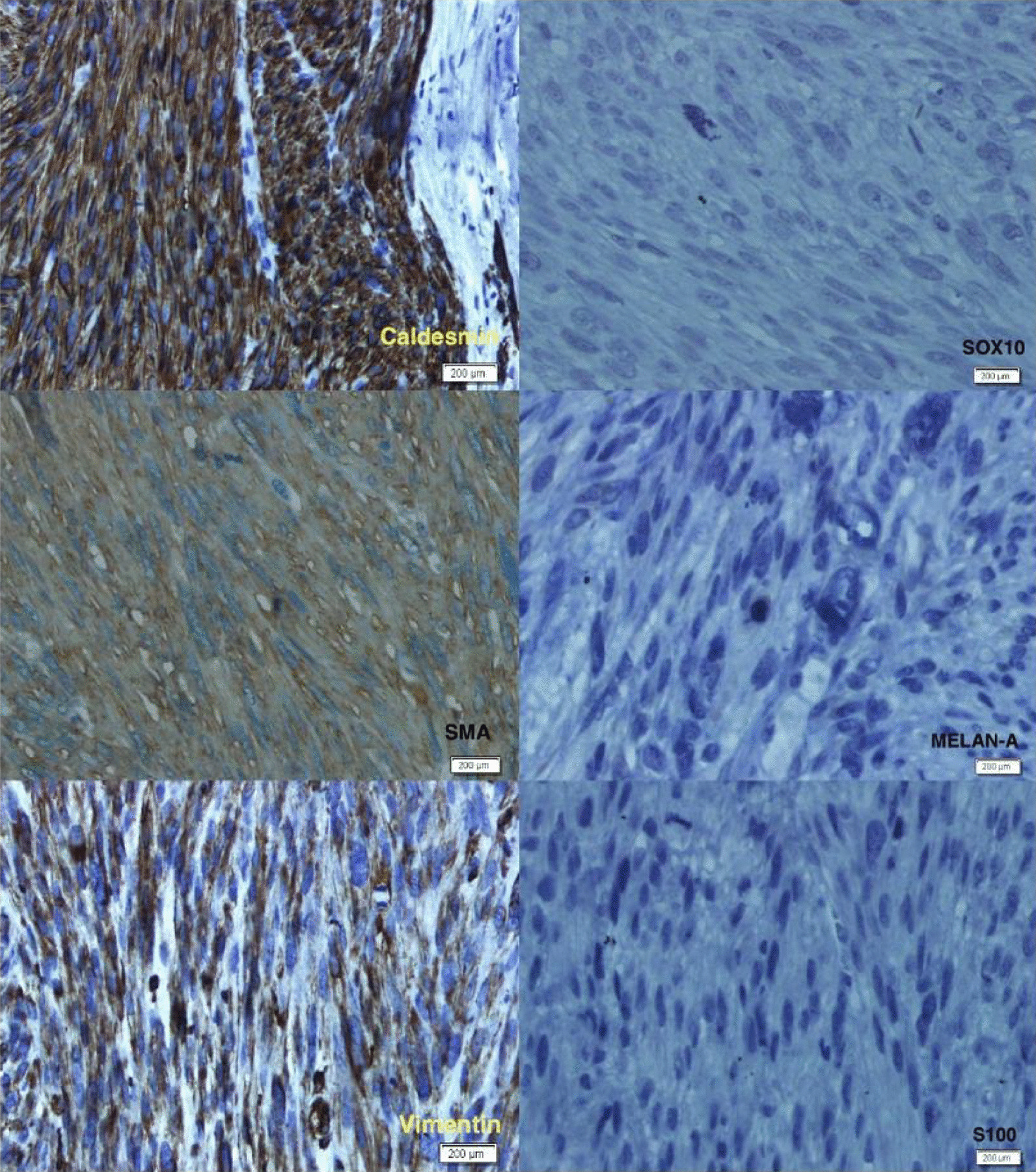
Immunohistochemistry study of the mass. Diffuse positivity for smooth muscle actin, desmin, caldesmon, and vimentin. Negative immunostaining for pan-cytokeratin, Melan A, SOX10, CD34, and S-100 markers

Histological study revealed an encapsulated nodular mass, composed of densely cellular atypical spindle cells with a fascicular arrangement. High power demonstrated spindle cells with cigar-shaped, prominently atypical nuclei. Multifocal necrosis with peripheral tumor cell palisading and occasional atypical mitotic figures (Fig. 3) was appreciated. According to the French Federation of Cancer Centers Sarcoma Group (FNCLCC) histologic grading system, the tumor differentiation score was 2, the mitotic count was about 10–19/10 high-power field (HPF) (score 2), and the tumor necrosis score was 2. The histological grade was reported as 3. Immunohistochemistry (IHC) staining showed diffuse positivity for smooth muscle actin (SMA), desmin, caldesmon, and vimentin, whereas immunostaining for pan-cytokeratin, Melan A, SOX10, CD34, and S-100 markers was negative (Fig. 4).

## Discussion

We described a rare case of malignant leiomyosarcoma arising from the scrotal wall in a 45-year-old man who was referred with a firm, nontender, mobile scrotal wall mass from 15 months before admission. Leiomyosarcoma of the scrotal wall is rare, and its clinical significance and prognosis have not been well defined. We reported the morphological features and immunohistochemical presentations of the tumor and its relatively long-term follow-up during which no evidence of recurrence or distant metastasis was discovered. Leiomyosarcomas have three typical histological features: perpendicularly arranged fascicles of spindle cells with eosinophilic cytoplasm, hyperchromatic blunt-ended nuclei, and scattered paranuclear vacuoles. Immunohistochemical staining shows an expression of SMA, muscle-specific actin, and desmin in most leiomyosarcomas, and expression of S-100 protein, CD34, Ki-67, myogenin, and cytokeratin has also been reported in some cases [5]. The French Federation of Cancer Centers Sarcoma Group (FNCLCC) grading system is based on the analysis of the number of mitoses (the mean number of mitoses in 5 HPF in a part of the tumor with the highest mitosis rate and cellularity), the severity of nuclear pleomorphism, and the percentage of necrosis [3]. These are important in predicting the biological behavior of the tumor (Table 1).

**Table 1:** The French Federation of Cancer Center Sarcoma Group grading syst﻿em

	Tumor differentiation	Mitotic count	Tumor necrosis
Score 0	–	–	No necrosis
Score 1	Sarcomas that closely resemble normal adult mesenchymal tissues	0–9 mitoses/10 HPF	< 50% tumor necrosis
Score 2	Sarcomas for which histologic typing is certain	10–19 mitoses/10 HPF	≥ 50% tumor necrosis
Score 3	Embryonal and undifferentiated sarcomas, synovial sarcoma, and sarcomas of uncertain differentiation	≥ 20 mitoses/10 HPF	–
Total score	2 or 3	Grade 1 (low grade)	
	4 or 5	Grade 2 (intermediate grade)	
	6, 7, or 8	Grade 3 (high grade)	

HPF: high-power field

We investigated Google Scholar for the peer-reviewed case reports of scrotal leiomyosarcomas from 1965 to 2021, and in Table 2 we have gathered all the reported cases that may clarify the disease and its management in patients for the future. The presentation depends on the site of the mass but is more likely presented as a painless, firm, slow-growing subcutaneous scrotal mass that is usually well defined, mobile, lobulated, and sometimes associated with a small hydrocele on palpation [3, 4].

**Table 2:** The disease management and outcome in the reported cases in the literature

Author	Age (year)	Treatment	F/U (months)	Outcome
Bouhout [7]	63	Mass excision	40	Survived
John [8]	73	Excision	9	Survived
Echenique [9]	80	Excision	12	Survived
Persichetti [10]	40	Mass excision	U/A	Unavailable
Dalton [11]	39	Wide local excision	18	Survived
Johnson [12]	56	Wide excision + RT	30	Survived + lung metastases
Ekmekcİ [13]	62	Wide local excision	48	Survived
Immergut [14]	38	Excision	24	Died—lymph node metastasis
Ray [15]	67	Local excision + after recurrence total excision	105	Died—groin metastases
Singh [16]	74	Mass excision	U/A	Unavailable
Planz [17]	85	Orchiectomy with high cord ligation	6	Survived
Singla [18]	60	Bilateral orchiectomy with high cord ligation	6	Survived
Talikoti [19]	67	Total scrotectomy and bilateral orchiectomy + RTx	19	Survived
Current case	45	Mass excision	20	Survived

F/U: Follow-up, RT: Radiotherapy, U/A: Unavailable

The primary imaging method for any scrotal abnormality is ultrasonography with a sensitivity of 95–100% for differentiating intratesticular from extratesticular lesions. A solid, heterogeneous, irregular mass with increased vascularity on color Doppler will usually be detected. It can also show variable echogenicity, with or without central necrosis or a whirling pattern; however, histological examination of tissue is required for diagnosis [4, 5]. Other imaging procedures like magnetic resonance imaging (MRI) or CT will be considered if needed. MRI will be used in assessing the stage by clarifying the local extent. CT of the chest, abdomen, and pelvis is the modality of choice for staging tumor spread and metastasis. CT and MRI can also be used to assess the presence of local, pelvic, or retroperitoneal lymphadenopathy, which can influence the surgical management [5]. However, the diagnostic gold standard for leiomyosarcoma is based on the histopathological examination of the specimen, which shows the spindle cells with cigar-shaped nuclei, and eosinophilic cytoplasm arranged in interlacing fascicles [6].

Distinguishing malignant and benign sarcomas is challenging, and simple excision of tumors is inadequate, as previous studies have indicated that up to one-third of patients who underwent simple excision had the residual microscopic disease [5]. The risk of local recurrence can increase if a positive margin is seen at the first excision. It is important to note that negative histological margins are tough to achieve during primary surgery [6, 9]. We performed a second, wider marginal excision of the tumoral bed aimed at the unquestionable surgical margins.

Earlier reports showed with surgery alone high rates of local failure will be seen; therefore, adjuvant radiotherapy is suggested for potential benefit in local control improvement. However, our patient did not agree with further treatment, so we placed him on closed clinical follow-up. The prognosis of leiomyosarcoma is related to its site, size, histological grade, and nodal or distant metastases [4]. The prognosis in the absence of local recurrence is almost always good. A positive margin at the first excision greatly increases the risk of local recurrence. Therefore, it is essential that the lesion is excised entirely and the sample sent for pathologic examination even for the most benign-looking scrotal mass. Because of local recurrence and distant metastasis even years after the initial excision, it is important to note the patients’ long-term follow-up [10].

## Conclusion

In summary, this case report outlines the morphological and immunohistochemical presentations of leiomyosarcoma of the scrotal wall and its long-term follow-up in a patient.

## Data Availability

Not applicable.
